# More than Just a Bag—Purple Urine Bag Syndrome as a Manifestation of Vulnerability in Geriatric Patients: A Case Report

**DOI:** 10.5811/cpcem.50688

**Published:** 2026-02-25

**Authors:** Lindsey White, Megan Rivera, Christopher J. Nash, Sreeja Natesan

**Affiliations:** Duke University Hospital, Department of Emergency Medicine, Durham, North Carolina

**Keywords:** purple urine bag syndrome, urinary catheter, social determinants of health, social vulnerability, case report

## Abstract

**Introduction:**

Purple urine bag syndrome (PUBS) is an uncommon yet visually striking condition observed in patients with long-term urinary catheters. It is associated with urinary tract infections caused by bacteria that metabolize tryptophan into indigo and indirubin pigments. Although typically benign, PUBS can signal underlying medical and social vulnerability.

**Case Report:**

We describe a 78-year-old woman with multiple sclerosis and chronic suprapubic catheterization who presented with failure to thrive and concerns for caregiver fatigue. A striking finding on arrival was the deep purple discoloration of her urine in the Foley bag, consistent with PUBS. Additionally, she was tachycardic and had extensive, unstageable pressure ulcers. Laboratory studies revealed leukocytosis, lactic acidosis, and acute kidney injury. Imaging suggested sacral osteomyelitis, stercoral colitis, and aspiration pneumonia. Blood cultures grew *Streptococcus dysgalactiae*, and she was treated empirically with broad-spectrum antibiotics. After goals-of-care discussions, she was transitioned to hospice and died shortly after discharge.

**Conclusion:**

While purple urine bag syndrome is often benign, its presence should prompt clinicians to evaluate for serious underlying disease, particularly in debilitated or high-risk patients. It is classically associated with chronic catheterization, alkaline urine, and infections involving organisms such as *Providencia stuartii*, *Klebsiella pneumoniae*, and *Proteus mirabilis*. This case highlights PUBS as a visible marker of potentially severe, multisystem pathology requiring timely and comprehensive assessment. Moreover, it underscores the role of social determinants of health such as inadequate home support, caregiver strain, and fragmented post-discharge care in exacerbating clinical decline. Recognition of these factors is essential for holistic care planning in frail older adults.

## INTRODUCTION

Purple urine bag syndrome (PUBS) is a rare phenomenon characterized by a purple discoloration of urinary catheter bags in patients with long-term indwelling catheters. 1 First described in 1978, PUBS is typically associated with urinary tract infections (UTI) involving bacteria that produce enzymes capable of metabolizing tryptophan into indigo (blue) and indirubin (red) pigments, resulting in the characteristic purple hue. 2 Although the urine appears purple in color, it is not the urine itself but the indigo and indirubin pigments coming into contact with the synthetic catheter tubing and bag that causes the discoloration. The urine may actually look typical of infected urine, which may range from normal to a dirty, cloudy appearance.

In addition to chronic catheterization, additional risk factors include female gender, constipation, and poor functional status. 3 Although alarming in appearance, PUBS itself is generally considered benign, but it can cause significant caregiver concern.

Although PUBS remains relatively rare, there are reports in the literature coinciding with a rise in the aging population. 1,4 As the incidence increases, PUBS highlights the broader systemic challenges in healthcare regarding our aging population, specifically increasing reliance on indwelling catheters and lack of adequate home health resources. 5–8 Inadequate home health can lead to suboptimal catheter care, delayed recognition of infection, caregiver fatigue, and repeated emergency department (ED) visits. In this case, a patient presented to the ED due to caregiver fatigue and was found to have PUBS, highlighting the ED’s evolving role as a safety net for patients whose complex care needs exceed what is currently available to them. It also emphasizes our opportunity to identify and intervene in cases. The management of PUBS in the ED is not just evaluation for underlying infection but also an opportunity to intervene in patients who need additional social and medical support.

## CASE REPORT

A 78-year-old female with a history of multiple sclerosis was brought to our ED via emergency medical services with concerns that her husband was unable to care for her at home. At baseline, she was mostly bed bound but able to transfer to a chair with assistance of a lift. She had not been eating or drinking well and had a worsening sacral ulcer. On obtaining further information from her husband, the patient had been at home for the prior five weeks after a seven-month admission to a skilled nursing facility due to complications from multiple sclerosis. He was having difficulty meeting her care needs in the home.

On arrival, the patient was found to have a suprapubic catheter with striking purple discoloration of the urine in the collection bag (Image). The patient and her husband were uncertain of the duration but confirmed that the catheter and bag had not been changed for over a month.

On exam, the patient was noted to be tachycardic, normotensive, and afebrile. Her breath sounds were clear bilaterally, and abdominal exam was soft and non-tender with a suprapubic catheter in place. She had multiple unstageable sacral and left lower extremity pressure ulcers tracking to bone. Her laboratory workup was remarkable for the following: lactate, 2.2 millimoles per liter (mmol/L) (reference range: 0.6–2.2 mmol/L); white blood cell (WBC) count of 21.7 x10^9/L (3.2–9.8x10^9/L), and evidence of acute kidney injury with a creatinine of 3.7 milligrams per deciliter (mg/dL) (0.4–1.0 mg/dL) (elevated from a baseline of 1.7 mg/dL). She was started empirically on broad-spectrum antibiotics with linezolid and piperacillin-tazobactam.

*CPC-EM Capsule*

What do we already know about this clinical entity?

*Purple urine bag syndrome (PUBS) is a rare, visually striking, benign discoloration in chronically catheterized patients caused by bacterial tryptophan metabolism*.

What makes this presentation of disease reportable?

*This case links PUBS with severe infection and caregiver fatigue, revealing social and medical vulnerability beyond its benign appearance*.

What is the major learning point?

*PUBS may appear harmless but should prompt evaluation for infection and assessment of home-care adequacy in frail patients*.

How might this improve emergency medicine practice?

*Recognizing PUBS can guide early infection workup and trigger social support interventions for at-risk, chronically ill patients*.

Her suprapubic catheter was exchanged in the ED, and a urine specimen was sent from the new catheter. Urinalysis was remarkable for greater than 182 WBCs per high powered field (hpf) (< 5/hpf) and 3+ leukocyte esterase (reference negative), and 3+ blood (reference negative). Computed tomography (CT) of the abdomen and pelvis without contrast revealed a large sacral decubitus ulcer with bony erosion suggestive of osteomyelitis, as well as findings consistent with stercoral colitis and a left lower lobe consolidation concerning for aspiration.

The patient was admitted to the general medicine service. Within the first two days, both sets of blood cultures grew *Streptococcus dysgalactiae*, while the urine culture grew mixed urogenital flora. On hospital day three, after goals-of-care discussion, the patient was transitioned to comfort care and was in the hospital for an additional 25 days for discharge planning. She was discharged home with hospice support and died 15 days post discharge.

## DISCUSSION

Purple urine bag syndrome is a rare but visually striking condition most often seen in elderly or functionally dependent patients with long-term urinary catheterization. While generally considered benign and not necessitating inpatient management, the increasing reports of PUBS observed in healthcare settings reflects broader health concerns, such as catheter-associated UTIs, inadequate catheter hygiene and, perhaps, poor attention to follow-up care.

In this case, the presence of PUBS was a visual clue that elucidated a patient’s clinical decline by insufficient home health support and caregiver fatigue. She carried multiple risk factors associated with PUBS, including chronic catheterization, female sex, poor functional status, and constipation. These are well-established contributors to PUBS, often representing polybacterial colonization and altered urinary tract physiology. 3 Although PUBS itself is not life-threatening, its presence should prompt an investigation for underlying infection. In this case, the patient was ultimately diagnosed with *S dysgalactiae* bacteremia and osteomyelitis, illustrating how PUBS should spur the investigation for underlying infections in this patient population.

The most common implicated bacterial pathogens are those that produce indoxyl sulfatase/phosphatase (Table). 9–13 The three most common organisms include *Escherichia coli* (20.8%), *Proteus mirabilis* (16.2%), and *Klebsiella pneumoniae* (13.6%). 10

Discolored urine has a broad differential diagnosis and includes a range of causes from metabolic conditions to medications and dietary factors. While the discoloration seen in PUBS is generally benign, it can be alarming to the patient and family. Laboratory evaluation should include a complete blood count, renal function testing (blood urea nitrogen, creatine and electrolytes), urinalysis, and urine culture. Management typically involves catheter exchange and treatment of underlying infection. Recurrence of PUBS may indicate incomplete treatment of a complicated UTI and should be addressed accordingly.

Beyond the clinical implications, PUBS brings attention to the care needs of aging and vulnerable patients, especially those transitioning from facility-based care to home settings. This patient returned home from a skilled nursing facility to an environment that was under-prepared for her needs with lack of professional support. This likely contributed to delayed recognition of infection and overall decline. It is suggested in the literature that delays in initiating home health services post-discharge are associated with increased rates of ED visits and hospital readmission. 6 Additionally, gaps in caregiver training, particularly around catheter care, may result in poor hygiene and missed signs of infection.

Today EDs are increasingly tasked with managing complex patients who present not only due to acute illness but also to challenges navigating the health system. Patients often present to the ED for social or logistical reasons, such as overwhelmed caregivers or lack of appropriate outpatient resources, rather than acute medical needs alone. Once in the ED, these patients often experience prolonged stays due to a lack of safe discharge plans, contributing to ED boarding. This case exemplifies how PUBS can serve as a visual indicator of such system-level challenges, underscoring the need for more comprehensive community-based care models that support these vulnerable populations while ensuring safe medical oversight.

## CONCLUSION

While purple urine bag syndrome itself may appear benign, it should prompt clinicians to consider broader questions, such as the presence of more serious underlying infection, patient safety, caregiver limitations, and systemic readiness to manage complex-needs patients in the outpatient setting. Recognizing these patterns is essential, as emergency clinicians are often the first to encounter signs of inadequate outpatient support. Strengthening access to home health services, equipping caregivers with appropriate education, and ensuring timely post-discharge follow-up are critical steps in reducing preventable ED use and improving outcomes in this vulnerable population.

## Figures and Tables

**Image:**
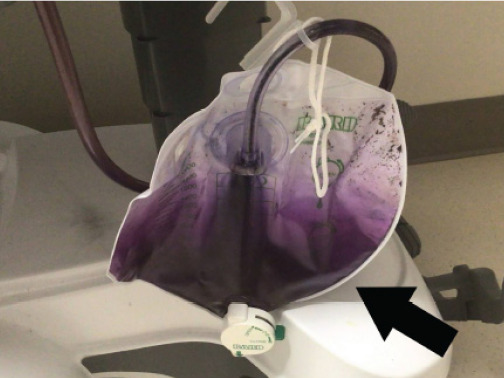
Photo depicting purple urine in catheter tubing and bag (arrow).

**Table:** Implicated pathogens in purple urine bag syndrome.

Enzyme Activity: Indoxyl sulfatase/phosphatase	
Most Common	*Enterococcus* spp. *Escherichia coli* *Klebsiella pneumoniae* *Morganella morganii* *Pseudomonas* genes *Proteus mirabilis* *Providencia stuartii* *Providencia rettgeri*
Less Common	*Citrobacter spp* *Staphylococcus spp*. *Streptococcus spp*. Methicillin-resistant *Staphylococcus aureus*.

*spp*, species.

## References

[1] Joseph J, Sundararaj JJ, Shekinah S (2025). Purple urine bag syndrome: case series. BMJ Support Palliat Care.

[2] Barlow GB, Dickson JAS (1978). Purple urine bags. Lancet.

[3] Al Montasir A, Al Mustaque A (2013). Purple urine bag syndrome. J Fam Med Prim Care.

[4] Ahmed SI, Waheed MA, Shah S (2022). Purple urine bag syndrome: a case report. Int J Surg Case Rep.

[5] Berendsen SA, van Doorn T, Blok BFM (2021). Urinary catheterization from 1997 to 2018: a Dutch population-based cohort. Ther Adv Urol.

[6] Topaz M, Barrón Y, Song J (2022). Risk of rehospitalization or emergency department visit is significantly higher for patients who receive their first home health care nursing visit later than 2 days after hospital discharge. J Am Med Dir Assoc.

[7] Smeekes OS, De Boer TR, Van Der Mei RD (2024). Receiving home care forms and the risk for emergency department visits in community-dwelling Dutch older adults, a retrospective cohort study using national data. BMC Public Health.

[8] Topaz M, Woo K, Ryvicker M (2020). Home healthcare clinical notes predict patient hospitalization and emergency department visits. Nurs Res.

[9] Yang H-W, Su Y-J (2018). Trends in the epidemiology of purple urine bag syndrome: a systematic review. Biomed Rep.

[10] Sabanis N, Paschou E, Papanikolaou P (2019). Purple urine bag syndrome: more than eyes can see. Curr Urol.

[11] Popović MB, Medić DD, Velicki RS (2023). Purple urine bag syndrome in a home-dwelling elderly female with lumbar compression fracture: a case report. Healthcare.

[12] Pereira AP, Camarinha I, Ferreira A (2024). Purple urine bag syndrome: a rare phenomenon managed in primary care. Cureus.

[13] Yaqub S, Mohkum S, Mukhtar KN (2013). Purple urine bag syndrome: a case report and review of literature. Indian J Nephrol.

